# Transportal versus all-inside techniques of anterior cruciate ligament reconstruction: a systematic review

**DOI:** 10.1186/s13018-021-02872-x

**Published:** 2021-12-23

**Authors:** Rohan Bhimani, Reza Shahriarirad, Keivan Ranjbar, Amirhossein Erfani, Soheil Ashkani-Esfahani

**Affiliations:** 1 Department of Orthopaedic Surgery, Massachusetts General Hospital, Harvard Medical School, Boston, USA; 2 Thoracic and Vascular Surgery Research Center, Shiraz University of Medical Science, Shiraz, Iran; 3 Student Research Committee, Shiraz University of Medical Sciences, Shiraz, Iran

**Keywords:** Anterior cruciate ligament, Anteromedial portal, All-inside technique, Single-bundle ACL reconstruction, Femoral tunnel, Tibial tunnel

## Abstract

**Background:**

Transportal (TP) and all-inside techniques (AIT) are the most commonly used anterior cruciate ligament (ACL) reconstruction procedures in current clinical practice. However, there is an ongoing debate over which procedure is superior. Therefore, the purpose of this systematic review was to evaluate and compare the clinical outcomes and complications of these two techniques to propose recommendations for future application. Our primary hypothesis was that AIT is a superior ACLR technique compared to TP.

**Methods:**

A systematic literature review, using PRISMA guidelines, was conducted using PubMed, Medline, Google Scholar, and EMBASE, up to February 2021 to identify studies focusing on AIT and TP techniques of ACL reconstruction. We excluded animal experiments, cadaveric studies, retrospective studies, case reports, technical notes, and studies without quantitative data. Patients’ characteristics, surgical technical features, along with postoperative follow-up and complications were extracted and reported accordingly. Methodological quality of the included studies was assessed using the Modified Coleman Methodology Score (MCMS).

**Results:**

A total of 44 studies were selected for this review, of which four were comparative studies. A total of 923 patients underwent AIT and 1678 patients underwent the TP technique for ACLR. A single semitendinosus graft was commonly used in the AIT compard to combined semitendinosus and gracilis graft in the TP group. The postoperative increase in International Knee Documentation Committee (IKDC), Lysholm, KT-1000, and Short Form-12 (physical and mental) scores were similar in the AIT group and the TP group. Contrastingly, the VAS pain score was significantly lower in the AIT group compared to the TP group. Furthermore, the pooled complication rates from all studies were similar between the two groups (AIT: 54 patients, 8.26% vs. PT: 55 patients, 6.62%). However, the four studies that prospectively compared AIT and TP techniques showed lesser complications in the AIT group than the TP group.

**Conclusion:**

Since the future trend in orthopedic surgery is toward less invasive and patients’ satisfaction with good outcomes, AIT is a good alternative method considering preserving bony tissue and gracilis tendon with less post-operative pain, along with more knee flexor strength and equal outcomes compared to conventional ACL reconstruction surgery.

*Level of Evidence* II.

**Supplementary Information:**

The online version contains supplementary material available at 10.1186/s13018-021-02872-x.

## Background

Anterior cruciate ligament (ACL) injury is common in athletes, with a female predominance [1, 2]. ACL deficit knee can result in high morbidity and long-term disability if inadequately treated [3]. The standard treatment for ACL injury is anterior cruciate ligament reconstruction (ACLR), which has evolved over time with the goal of achieving a more anatomical and less invasive reconstruction method because previous non-anatomic repairs were shown to have a higher risk of graft impingement, rotational instability, and graft attenuation [4–8]. The transportal (TP) and all-inside techniques (AIT) are the most commonly used reconstruction procedures in current clinical practice.

TP technique is a popular and widely practiced technique of ACLR, as it allows independent femoral tunnel drilling [9, 10]. Among the advantages of this technique is it does not require special equipment, performance ease, and its ability to reach the center of the native ACL footprint [11]. However, a caveat to this technique is that it may result in disproportionate stress on the graft which increases the possibility of graft failure, rupture of the femoral posterior wall, and short femoral tunnel length [10, 12–17]. Robin et al. in a review reported other shortcomings of TP technique such as difficulty visualizing in hyperflexion possibly leading to iatrogenic chondral injury, technically demanding, short or bicortical sockets—which may limit fixation options, higher revision rate, increased risk of injury to the common peroneal nerve, and extension loss during stance phase [18]. Furthermore, hyperflexion requires an assistant, thus entails for improving and developing better techniques [18].

AIT has been acclaimed to be an alternative to the TP technique [19]. It uses sockets in a half-way tunnel rather than full tunnels, resulting in a reduction in the post-operative pain, swelling, and likelihood of synovial fluid flow or infiltration among the space between the graft and the bone interface [20, 21]. Furthermore, the sockets can also prevent tunnel enlargement and accelerate graft maturation due to the eradication of dead space [22]. Among the other advantages are the made small incision from a cosmetic aspect [22], less invasiveness and variety of graft choices [23]. However, AIT is associated with learning curve and increased risk of injuring extra-articular surface. Based on the aforementioned benefits and drawbacks, AIT is now considered a new option for ACL reconstruction.

While prior studies have demonstrated the utility of AIT and TP technique, fewer have elucidated superiority of one technique over the other in terms of clinical outcome [24]. Our primary hypothesis was that AIT is a superior ACLR technique compared to TP, therefore, in this review, we aimed to evaluate the available data in the literature in terms of outcome and complications of these two techniques to propose recommendations for future application. AIT is a superior ACLR technique compared to TP.

## Materials and methods

### Search strategy

Four major online databases (EMBASE, PUBMED, MEDLINE, and Google Scholar) were screened for the related literature addressing ACLR methods. Articles that were published until February 6, 2021, were enrolled. The keywords used in this study were based on MeSH terms and included “anterior cruciate ligament reconstruction,” and similar phrases (Additional file 1: Table S1). Our search method was focused on the AIT and TP techniques of ACLR. In our study, the AIT was defined as creating the bone socket from the articular side of the tibia rather than the traditional full-length tunneling through the knee joint and outer cortex. [20, 21]. Due to the anticipated scarcity of published literature, the search was not limited to randomized controlled trials.

### Study selection

We included clinical studies involving individuals ≥ 18 years old, articles written in English, and surgeries limited to primary ACLR or where ACLR was the primary purpose of the surgery. We excluded animal experiments, cadaveric studies, retrospective studies, case reports, technical notes, and studies without quantitative data. Furthermore, in studies with mixed populations or various techniques, only data regarding our inclusion criteria (AIT or TP) were extracted for the data analysis. Since there were no readily available criteria for anatomic ACLR, we have chosen to include all articles in which the authors stated that the reconstructive surgical procedure they performed was the AIT or TP techniques, or that the described technique used in their study indicated the use of AIT or TP.

### Data extraction, quality assessment and analysis

Three reviewers screened all the selected literature independently. First of all, the abstracts were reviewed, and if the content of the abstract revealed the relevance of the results of the paper to our aims full texts would be taken into consideration. Disagreements on including or excluding the papers or interpreting the outcomes of the studies were discussed among the reviewers and resolved. The reviewers independently assessed the quality of included studies using the Coleman Methodology Score (CMS) [25]. The score is based on ten subsections derived from the CONSORT statement for randomized controlled trials. The total score is between 0 and 100. A score of 100 indicates that the study largely avoids chance, various biases, and confounding factors. A worksheet for data extraction was created and used to obtain a descriptive review of the reported variety of surgical and demographic data from each study (Additional file 2: Table S2). Recorded data included study characteristics (author, year of publication, sample size, and study design), descriptive statistics, and clinical data. Descriptive statistics such as means, mean differences, standard deviation (SD), and measures of variance are presented where applicable. Means of ranges are presented where distributions of data were unavailable. A pooling method of means and variances was utilized to calculate the overall outcome scores.

## Results

### Study characteristics

**Fig. 1:**
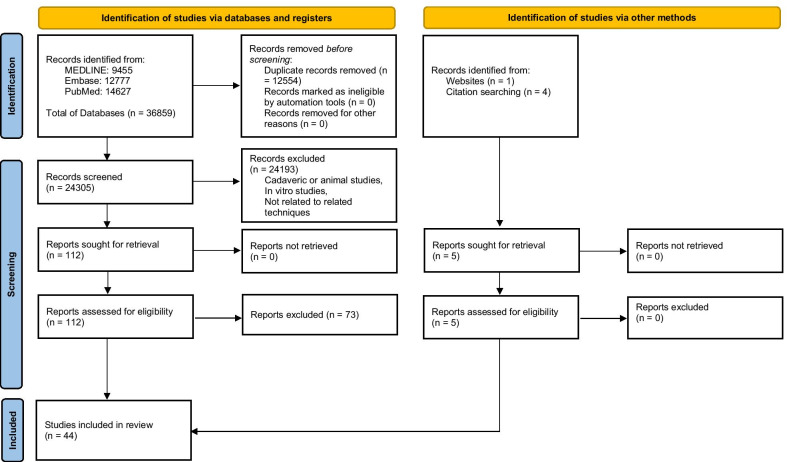
PRISMA Flowchart of included articles regarding all-inside and transportal anterior cruciate ligament reconstruction

The initial search yielded a total of 36,859 articles. After excluding 12,554 duplicates, a systematic screening process ultimately yielded 44 articles, 15 full-text articles regarding AIT [26–40], 25 regarding TP technique [41–65], and four [66–69] articles regarding AIT versus TP technique which were included in this review (Fig. 1). The demographic data of the patients who underwent AIT are shown in Table 1, while patients who underwent TP are demonstrated in Table 2. Also, the comparison of the two techniques is presented in Table 3.

**Table 1:** Review of literature on all studies regarding All-inside reconstruction of the anterior cruciate ligament

References	No of patients (male/female)	Study design	CMS	Mean age (± SD)	Graft type	Fixation technique		Subjective IKDC score change (follow-up)	Lysholm score change (follow-up)	Lachman test [score post-op] (follow-ups)
						Tibial	Femoral			
Yasen et al. [39]	108 (81/27)	Prospective	85	30.9	ST	Cortical suspensory fixation	Cortical suspensory fixation	–	33.2 (2 year), 31.4 (1 year)	–
Otsuka et al. [33]	20 (8/12)	Clinical trial	89	21.1	BPTB	Metal interference screws	Metal interference screws	–	–	–
Schurz et al. [35]	79 (52/27)	Prospective	85	29	ST	Cortical suspensory fixation	Cortical suspensory fixation	45.1 ± 13.29 (2 year), 44.9 ± 11.1 (1 year)	39.7 (2 year), 37.7 (1 year)	–
Shah et al. [36]	40 (36/4)	Prospective	74	27.1	ST	Cortical suspensory fixation	Cortical suspensory fixation	–	23.8 ± 19.42 (1 year)	All [normal] (1 year)
Lubowitz et al. [30]	27 (9/18)	Randomized controlled trial	80	41.6	Posterior TT	Tibial Retro Screw (Arthrex) (Aperture)	Bio-Composite femoral interference screw (Aperture)	33.2 ± 16.28 (2 year), 33.5 ± 17.44 (1 year)	–	–
	31 (11/20)	Randomized controlled trial	80	40.2	Posterior TT	Titanium cortical button (Arthrex) (Suspensory)	Femoral fixed loop length Retro-Button (Arthrex) (Suspensory)	36.9 ± 21.83 (2 year), 33.2 ± 20.08 (1 year)	–	–
Volpi et al. [37]	20 (12/8)	Controlled trial	71	38.4 ± 10.8	ST	Metallic cortical suture button	Cortical femoral fixation	–	–	–
Dujardin et al. [29]	20 (12/8)	Prospective	61	28 ± 8.2	ST (anterior)	–	–	–	–	–
	19 (10/6)	Prospective	61	24 ± 6.7	ST (posterior)	–	–	–	–	–
Buda et al. [34]	31 (31/0)	Prospective	74	27 ± 8.7	ST	–	–	36.4 ± 6.84 (1 year)	–	30 [normal]; 1 [mild instability] (1 year)
Monaco et al. [31]	22 (15/7)	Controlled trial, III	86	32.5 ± 6.7	ST	Flip-then-fill technique	Flip-then-fill technique	41 (2 year)	36 (2 year)	–
Bi et al. [26]	62 (34/28)	Randomized controlled trial	95	29.1 ± 6.5	AHPLT	Tightrope	Tightrope	36.7 ± 10.32 (2 year)	–	–
	62 (31/31)	Randomized controlled trial	95	27.9 ± 6.7	ST	Tightrope	Tightrope	39.2 ± 9.23 (2 year)	–	–
Watanabe et al. [38]	24 (13/11)	Prospective, IV	79	31.0	ST	Endo Button	Endo Button CL BTB	–	39 ± 15.23 (2 year)	All [normal]
Blackman and Blackman [27]	95 (60/35)	Prospective	69	27.6	ST, GT	Suspensory button	Suspensory button	–	–	–
Bressy et al. [28]	35 (22/13)	Prospective	66	27 ± 7.8	ST	Suspensory button	Suspensory button	29.8 ± 26.83 (> 1 year)	35.3 ± 30.71	–
Nawabi et al. [32]	23 (15/8)	Prospective	66	12.6 ± 1.25	ST	Attachable button system (ABS) (Tightrope)	Tightrope (reverse tensioning button)	–	–	–
Benea et al. [66]	23 (16/7)	Prospective Randomized	74	28.4 ± 8.6	ST	Suture button	Tight rope	20.7 ± 20.58 (6 months)	–	–
Brandsson et al. [40]	29 (20/9)	Prospective Randomized	87	27	BPTB	Interference screw rear entry	Interference screw rear entry	–	20 ± 22.67	–
Kouloumentas et al. [67]	45 (28/17)	Prospective Randomized, I	87	27.6 ± 11.4	ST	Tight rope (suspensory fixation)	Tight rope (suspensory fixation)	41.7 ± 15.12 (2 year)	52.1 ± 15.84	–
Lubowitz et al. [68]	76 (38/37)	Prospective Randomized, 1	87	39.3 ± 12.1	TT	Bio-absorbable tibial interference screw	Bio-absorbable femoral interference screw (Arthrex, Naples, FL)	39.1 ± 18.96 (2 year)	–	–
Russu et al. [69]	32	Prospective Randomized	77	–	ST	–	–	19.76 ± 16.61 (6 months)	–	–

*CMS* Coleman methodology score, *ST* semitendinosus, *BPTB* bone patellar tibial bone, *TT* tibial tendon, *GT* gracilis tendon, *AHPLT* anterior half of peroneus longus tendon

**Table 2:** Review of literature on all studies regarding transportal reconstruction of the anterior cruciate ligament

References	No of patients (male/female)	Study design	CMS	Mean age (± SD)	Graft type	Fixation technique		Subjective IKDC score change (follow-up)	Lysholm score change (follow-up)	Lachman test [score post-op] (follow-ups)
						Tibial	Femoral			
Kim et al. [50]	53 (40/13)	Randomized controlled trial	92	36.4 ± 10.1	HT, Anterior TT	Bio-absorbable interference screw	Endo Button	33.6 ± 18.55 (2 year)	23.2 ± 23.76 (2 year)	45[0]; 5[+ 1]; 2[+ 2]; 1[+ 3] (2 year)
Clockaerts et al. [43]	16 (9/7)	Randomized controlled trial	70	34.4 ± 10.0	Ipsilateral HT	–	–	–	–	–
Lee et al. [55]	31 (21/8)	Randomized controlled trial	56	32.0 ± 8.3	ST for AM bundle and GT for PL bundle	bioabsorbable interfer-ence screws	cortical suspension system	–	–	–
Guglielmetti et al. [45]	38 (NA)	Randomized controlled trial	77	24	GT, ST	A metal interference screw	Endo Tunnel Device (ETD®	–	–	29 [0]; 9 [+ 1]; 0 [+ 2]; 0 [+ 3]; (6 months)
Noh et al. [59]	31 (31/0)	Randomized controlled trial	79	22	fresh-frozen AT	Bio-interference screw	EndoButton CL	–	39 (2 year)	25[0]; 5 [+ 1]; 1 [+ 2]; 0 [+ 3] (2 year)
Kyung et al. [54]	38 (28/10)	Randomized controlled trial	56	37.4 ± 11.4	ST for AM bundle and GT for PL bundle	bioabsorbable interference screws with a post tie	cortical suspensory device	–	–	-
Koutras et al. [53]	15 (15/0)	Prospective non-randomized trial	64	21.5 ± 4	HT	Cross-pins or endobutton	Cross-pins or endobutton	–	–	–
Kim et al. [49]	40 (34/6)	Prospective randomized controlled trial	66	36.5 ± 10.1	ST for AM bundle and GT for PL bundle	suspensory fixation	suspensory fixation	–	–	–
Kim et al. [51]*	40 (34/6)	Prospective randomized controlled trial	66	36.5 ± 10.1	ST for AM bundle and GT for PL bundle	suspensory fixation	suspensory fixation	–	–	–
Mirzatolooei [58]	80 (79/1)	Randomized controlled trial	77	26.6	HT	Cross-pin fixation using a TransFix®	Cross-pin fixation using a TransFix®	–	–	70 [negative]; 10 [positive] (2 year)
Kim et al. [52]	21 (18/3)	Randomized controlled trial	68	36.7 ± 10.3	ST for AM bundle and GT for PL bundle	Bioabsorbable interference screw	EndoButtonCL (Smith & Nephew Endoscopy)	–	–	–
Fujita et al. [44]	18 (6/12)	Prospective randomized study	89	26.9	ST, GT	ndoButtonCL and a post screw	ndoButtonCL and a post screw	–	30 ± 6.74 (2 year)	–
Clatworthy et al. [42]	464 (297/167)	Prospective comparative study	44	32.3	HT	Intra tendon tibial screw and sheath device (Mitek, Intrafix or Arthrex, Graftbolt)	Suspensory devices (Smith and Nephew, Endobut-ton Continuous Loop or Arthrex, Retrobutton RT)	–	–	–
Youm et al. [64]	20 (19/1)	Randomized controlled trial	76	27.6 ± 9.9	Fresh-frozen AT	Bioabsorbable interference screw	Metal interference screw	–	–	18 [negative] 2 [positive] (2 year)
Bohn et al. [41]	12 (NA)	Prospective randomized clinical trial	74	24.3 ± 4.9	ST, GT	Biodegradable interference screw	EndoButton CL	14 ± 17.03 (1 year)	13 ± 18.44(1 year)	72% [normal] (1 year)
Pujol et al. [61]	29 (16/13)	Prospective randomized single-blind	74	31.24	HT, BPTB	11 interference screw,18 double fixation	17 interference screw,12 cortical button	30/35 (1 year)	20/96 (1 year)	–
	25 (17/8)	Prospective randomized single-blind	74	28.56	HT, BPTB	8 interference screw, 17 double fixation	12 interference screw,13 cortical buttion	28/93 (1 year)	20/78 (1 year)	–
Rezazadeh. et al. [62]	50 (45/5)	Prospective	69	30.6 ± 6.5	–	–	–	–	–	48 [0];1 [+ 1]; 1[+ 2]; 0[+ 3] (1 year)
Silva et al. [63]	20 (NA)	Prospective	40	24 ± 5.2	ST, GT	Bioabsorbable interference screw	Toggleloc Ziploop (BiometTM)	–	–	–
Maestro et al. [57]	26 (NA)	Prospective	40	28.6 ± 6.4	ST	Bioabsorbable interference screw	Endobutton CL cortical suspensorry	–	–	–
	13 (NA)	Prospective	40	27.3 ± 6.9	ST	Bioabsorbable interference screw	Endobutton CL cortical suspensorry	–	–	–
Özer et al. [60]	30 (28/2)	Nonrandomized prospective trial	74	28.07 ± 7.42	ST, GT	Interference screw	AO screw	31.8 ± 13.74 (1 year)	24.98 ± 10.07 (1 year)	24 [0]; 6 [+ 1]; 0[+ 2]; 0[+ 3] (1 year)
Hussin et al. [47]	30 (NA)	Prospective single-blinded randomized controlled trial	74	–	ST, GT	–	–	39 (1 year)	35 (1 year)	–
Karikis et al. [48]	49 (31/18)	Prospective	82	32 ± 8.8	HT	interference screw	interference screw	–	18.2 ± 24.15 (2 year)	–
	45 (32/13)	Prospective	82	29.6 ± 8.4	ST, GT	Bioresorbable screws	Metal interference screw	–	22.1 ± 22.96 (2 year)	–
MacDonald et al. [56]	46 (31/15)	Single-blinded, prospective, randomized	77	30.7 ± 9.3	ST, GT	Biocomposite interference screw	Cortical suspensory button	41 (2 year)	–	–
Zhang et al. [65]	38 (NA)	Prospective randomized single-blind	77	–	ST, GT	Intrafix system	Rigidfix system	–	28.4 ± 5.3 (1 year)	–
Hussein et al. [46]	78 (46/32)	Prospective randomized	87	34.2	ST, GT	Bioabsorbable interference screw	EndoButton	22.9 ± 15.39 (3- to 5-Year)	18.5 ± 13.5 (3- to 5-Year)	–
	131 (68/63)	Prospective randomized	87	32.3	ST, GT	Bioabsorbable interference screw	EndoButton	25.1 ± 15.88 (3- to 5-Year)	20 ± 13.03 (3- to 5-Year)	–
Benea et al. [66]	23 (13/10)	Prospective Randomized study	74	30.2 ± 9.4	ST, GT	Interference screw	Interference screw	18.6 ± 21.33 (6 months)	–	–
Kouloumentas et al. [67]	45 (27/18)	Prospective Randomized study	87	29.7 ± 11.0	ST, GT	Interfrenece screw (Megafix® absorbable)	Suspensory fixation (cortical button) Flipptack™ button system	34.9 ± 17.15 (2 year)	51.8 ± 17.63 (2 year)	–
Lubowitz et al. [68]	72 (39/34)	Prospective Randomized study	87	41.1-+ 10.8	Anterior TT	Bioabsorbable tibial interference screw	Bioabsorbable tibial interference screw	34.4 ± 20.38 (2 year)	–	–
Russu et al. [69]	31 (NA)	Prospective Randomized study	77	–	ST, GT	–	–	22.88 ± 15.44 (6 months)	–	–

*CMS* Coleman methodology score, *ST* semitendinosus, *BPTB* bone patellar tibial bone, *TT* tibial tendon, *GT* gracilis tendon, *HT* hamstring tendon, *AHPLT* anterior half of peroneus longus tendon

*Same dataset was used in two separated articles

**Table 3:** Summary of data in literature regarding All inside technique (AIT) and transportal (TP) technique in anterior cruciate ligament reconstruction

Variables	AIT *n* = 923	TP *n* = 1678
Gender; n(%)		
*Male*	557 (62.58%)	846 (73.63%)
* Female*	333 (37.42%)	303 (26.37%)
Age (years); mean (SD)	30.06 (6.21)	31.54 (5.82)
Population of study type of injury; n(%)		
*Isolated*	131 (48.52%)	31 (20.53%)
*Concomitant*	139 (51.48%)	95 (62.91%)
*Complete ACL rupture*	–	25 (16.56%)
Interval between time of injury till surgery (weeks); mean (SD)	23.96 (14.07)	76.65 (32.1)
Average follow-up post-surgery (weeks); mean (SD)	31.11 (1.78)	26.7 (5.18)
Modifications in technique	All epiphyseal AIT: 15 (6.98%) Double-bundle AIT: 24 (11.16%) Trans-lateral: 148 (68.84%) Transtibial: 20 (9.3%) Partial- transphyseal: 8 (3.72%)	Single bundle: 302 (49%) Double bundle: 314 (50.97%)
Graft type	Semitendinosus: 664 (81.47%) Tibialis anterior tendon: 134 (16.44%)	Semitendinosus and gracilis: 768 (49.83%) Hamstring: 670 (43.48%) Achilles: 51 (3.3%)
Graft source; n(%)		
*Autograft*	598 (76.67%)	1063 (84.3%)
*Allograft*	182 (23.33%)	198 (15.7%)
Spinning; n(%)		
*Double*	134 (13.97%)	103 (29.5%)
*Quadruple*	712 (74.24%)	83 (23.78%)
*Six-strand*	–	163 (46.7%)
*8-strand*	113 (11.78%)	–
Drilling technique; n(%)		
*Femoral*	Inside out: 433 (48%) Anteromedial: 177 (19.62) Outside-in: 101 (11.19%) Retro-drill: 109 (12.08%) Anterograde/retrograde: 82 (9.09%)	Offset guide: 16 (14.41%) Inside out complete tunnel: 23 (20.72%) Anteromedial portal technique: 72 (64.86%)
*Tibial*	Inside-out: 475 (52.66%)	Tibial guide: 74
	Outside in: 79 (8.75%)	Outside in: 23
	Retro drill: 348 (38.58%)	Antegrade cannulated drilling: 72
Socket and fixation; (mm)		
*Range Femoral/Tibial*	20–25/20–35	35.5–39.9/–
*Average Femoral/Tibial*	20.62/ 31.77	38.74 (0.27)/–
Rehabilitation; mean (SD; range)		
*Return to sports* (months)	7 (1.73; 4–12.5)	8.3 (3.23; 6–12)
Complications; n(%)		
Total	54 (8.26%)	55 (6.62%)
* Graft failure*	14 (25.93%)	5 (9.09%)
* ACL failure*	10 (18.25%)	36 (65.45%)
* Paresthesia*	9 (16.67%)	
*Re-operation*	9 (16.67%)	
*Meniscus injury*	6 (11.11)	
* Septic arthritis*	3 (5.5%)	
* Superficial infection*	3 (5.5%)	
*Deep infection*	3 (5.5%)	
*Hypoesthesia*	2 (3.7%)	
*Neurapraxia*	2 (3.7%)	
*Hemarthrosis*	2 (3.7%)	1 (1.82%)
* Cyclops syndrome*	2 (3.7%)	3 (5.45%)
*Wound dehiscence*	1 (1.85%)	
*Flexion loss*	1 (1.85%)	
*Arthrofibrosis*	1 (1.85%)	2 (3.64%)
*Superficial hematoma*	1 (1.85%)	1 (1.82%)

ACL: Anterior Cruciate Ligament; SD: Standard Deviation

### All-inside technique

Among 19 articles regarding AIT [26–40, 66–69] a total of 923 cases of ACLR treated with AIT, of which their regarding data are exhibited in Tabled 1 and 3. The type of graft was mentioned in 815 patients; in 664 (81.47%) patients semitendinosus tendon was used. In 598 patients (76.67%) autograft was used. The average length and the diameter of grafts used were 63.58 ± 3.69 and 8.27 ± 0.65, respectively. Among the fixation techniques, suspensory fixation was a preferred choice as reported in 821 patients (Table 1).

### Transportal technique

Based on the report of 29 articles [41–69], a total of 1678 patients were treated with the TP technique, of which the regarding data are exhibited in Tables 2 and 3. The type of graft used was mentioned in 1541 cases, 768 (49.83%) used a combination of semitendinosus and gracilis tendons. Autograft was used in 1063 patients (84.3%) while allograft was used in 198 patients (15.7%). The interference screw was the preferred fixation method used in 806 patients (52.64%) for the tibial side and 311 patients (20.31%) for the femoral side (Table 2).

### All-inside versus transportal

Among the studies included in our review, four prospective randomized studies [66–69] have compared the outcomes of the AIT to TP technique (Table 4), which included 176 AIT and 171 TP patients. The male to female ratio in these studies were 82:61 and 79:62 for AIT and TP, respectively. The average age in the groups were 27.73 ± 10.32 years and 29.18 ± 9.65 years for AIT and TP, respectively and the average BMI in the AIT groups was 25.8 ± 5.05 kg/m^2^ and in the TP group was 24.29 ± 2.74 kg/m^2^.

**Table 4:** Review of literature regarding all-inside reconstruction of the anterior cruciate ligament versus transportal reconstruction technique

References	Number of patients	Gender (male:female)	Graft type	Age (mean ± SD)	Method of reconstruction	Drilling technique		Graft fixation technique		Scoring system		Duration follow-up
						Drilling femoral	Drilling tibial	Fixation femoral	Fixation tibial	Pre-op score (Mean ± SD)	Post op score (Mean ± SD)	
Benea et al. [66]	23	16:7	ST	28.4 ± 8.6	All inside	Antro medial portal (in–out)	Retrodrilling (in–out)	Tight rope	Suture button	60.6 ± 14.7 (IKDC)	81.3 ± 14.4 (IKDC)	2 years
										30.9 ± 20.1 (VAS of 100 mm)	0.9 ± 1.5 (VAS of 100 mm)	
	23	13:10	ST and GT	30.2 ± 9.4	Classical	Inside out complete tunnel	Outside in complete tunnel	Interference screw	Interference screw	62.5 ± 13.4 (IKDC)	81.1 ± 16.6 (IKDC)	
										28 ± 20.3 (VAS of 100 mm)	4.1 ± 9.4 (VAS of 100 mm)	
Kouloumentas et al. [67]	45	28:17	ST	27.6 ± 11.4	All inside	–	–	TightRope (suspensory fixation)	TightRope (suspensory fixation)	68.6 ± 6.6 (KOOS)	95.3 ± 3.8 (KOOS)	2 years
										41.9 ± 12.7 (IKDC)	83.6 ± 8.2 (IKDC)	
										45.6 ± 15.7 (Lysholm)	97.7 ± 2.1 (Lysholm)	
										54.8 ± 15.6 (KSS score)	83.9 ± 11.8 (KSS score)	
	45	27:18	ST and GT	29.7 ± 11.0	Conventional	–	–	Suspensory fixation (cortical button) Flipptack™ button system	Interfrenece screw (Megafix® absorbable)	65.9 ± 7.2 (KOOS)	95.8 ± 3.6 (KOOS)	
										43.6 ± 14 (IKDC)	78.5 ± 9.9 (IKDC)	
										44.8 ± 17.5 (Lysholm)	96.6 ± 2.2 (Lysholm)	
										58.4 ± 17.4 (KSS score)	96.6 ± 2.8 (KSS score)	
Lubowitz et al. [68]	76	38:37	Anterior TT	39.312.1	All inside	Anteromedial portal technique	Retrograde drilling technique (RetroDrill: Arthrex)	Bioabsorbable femoral interference screw (Arthrex, Naples, FL)	Bioabsorbable tibial interference screw	47.4 ± 15.0 (IKDC)	86.5 ± 11.6 (IKDC)	2 years
										2.6 ± 2.1 (VAS score)	− 2.5 ± 2.0 (VAS score)	
										37.5 ± 9.6 (SF-12 Physical)	53.3 ± 6.6 (SF-12 Physical)	
										54.6 ± 9.6 (SF-12 Mental)	56.8 ± 3.8 (SF-12 Mental)	
										68.9 ± 17.2 (KSS- Pain)	93.3 ± 15.1 (KSS- Pain)	
										61.3 ± 30.1 (KSS- Function)	97.6 ± 6.7 (KSS- Function)	
	72	39:34	Anterior TT	41.110.8	Full tibial tunnel	Anteromedial portal technique	Antegrade, cannulated drilling technique	Bioabsorbable femoral interference screw (Arthrex, Naples, FL)	Bioabsorbable tibial interference screw	49.6 ± 16.4 (IKDC)	84.0 ± 12.1 (IKDC)	
										1.6 ± 2.0 (VAS score)	-1.7 ± 2.1 (VAS score)	
										38.9 ± 9.4 (SF-12 Physical)	52.5 ± 6.9 (SF-12 Physical)	
										53.3 ± 10.6 (SF-12 Mental)	55.3 ± 6.7 (SF-12 Mental)	
										73.2 ± 16.3 (KSS- Pain)	95.9 ± 7.4(KSS- Pain)	
										60.3 ± 30.4 (KSS- Function)	98.8 ± 5.0 (KSS- Function)	
Russu et al. [69]	32	–	ST	–	All inside	–	–	–	–	52.48 ± 10.24 (KOOS)	83.45 ± 9.58 (KOOS)	6 months
										60.2 ± 16.61 (IKDC)	79.96 ± 10.78 (IKDC)	
										2.4 ± 0.9 (VAS score)	-1.5 ± 1.9 (VAS score)	
	31	–	ST and GT	–	Full tibial tunnel	–	–	–	–	53.81 ± 12.60 (KOOS)	82.68 ± 8.68 (KOOS)	
										58.32 ± 12.85 (IKDC)	81.20 ± 8.56 (IKDC)	
										2.3 ± 0.7 (VAS score)	-1.3 ± 2.9 (VAS score)	
										2 ± 0.90 (Tegner score)	6 ± 0.88 (Tegner score)	

*SD* standard deviation, *ST* semitendinosus, *TT* tibial tendon, *GT* gracilis tendon, *IKDC* international knee documentation committee, *KOOS* knee injury and osteoarthritis outcome score, *VAS* visual analog scale, *KSS* knee society score

In terms of graft characteristics, a combination of semitendinosus and gracilis tendon (49.8%) was the preferred graft in TP patients and isolated semitendinosus in AIT patients (81.5%). The mean graft diameter in AIT was 8.2 ± 0.7 mm for the femoral side and 8.3 ± 5 mm for the tibial side while for TP the mentioned values were 7.7 ± 0.5 mm and 7.7 ± 4.9 mm for femoral and tibial side, respectively [67]. Among four studies, two of them used allograft [67, 68] for both techniques while the remaining two used autografts [66, 69]. In other words, 68.42% of TP and 66.48% of AIT grafts were allografts. In addition, one study used a quadruple bundle for ACLR [67] while another study used a double bundle for ACLR [68].

### Physical examination and functional outcome scores

The postoperative outcome scoring system varied among the studies and is summarized in Table 5. Among 153 AIT-ACLR patients, 145 (94.77%) had a normal pivot shift test, while eight (5.22%) had positive tests. Similarly, among 686 TP ACLR patients, 595 (86.73%) had normal pivot shift test while 93 (13.27%) had abnormal test results. Furthermore, based on the Knee Society Scoring system [40] in AIT-ACLR patients, an increase of 24.29 ± 20.27 for pain and 31.31 ± 27.17 for function during a two year follow-up was observed, while these measures were 20.84 ± 18.75 and 29.16 ± 26.32 for pain and function, respectively, during a one-year follow-up. Furthermore, when compared to the preoperative score at two years follow-up, the postoperative Lysholm score increased by 37.13 and 27.99 points in the AIT and TP groups, respectively. Moreover, no significant difference was seen in IKDC, KSS and KOOS scores between the two groups (Tables 4, 5).

**Table 5:** Change of scores among All-inside and transportal anterior cruciate ligament reconstruction based on scoring method and follow-up duration

Scoring system	Technique	Change of score during follow-up			
		6 months	1 year	2 years	3–5 years
KOOS	AIT	+ 30.97 (14.02)	+ 28.1	+ 29.97 (22.71)	–
	TP	+ 28.87 (15.3)	+ 20.68 (7.39)	+ 29.9 (8.05)	–
IKDC	AIT	+ 28.04 (16.42)	+ 40.58 (13.13)	+ 38.99 (14.41)	–
	TP	+ 25.8 (16.49)	+ 34.47 (6.01)	+ 34.09 (15.39)	+ 24.28 (15.7)
KT-1000	AIT	− 5.5 (0.9)	− 4.34 (1.947)	− 3.94 (2.15)	–
	TP	–	− 1.8 (4.41)	− 2.87 (3.5)	–
Lysholm	AIT		+ 31.49 (9.95)	+ 37.13 (10.48)	–
	TP	+ 28 (10.83)	+ 28.76 (6.16)	+ 27.99 (18.46)	+ 19.44 (13.2)
VAS	AIT	–	− 5.54 (1.15)	− 3.56 (2.14)	–
	TP	− 12.25(14.72)	–	− 3.3 (2.9)	–
Short form-12 scoring system	AIT	Physical: + 13.6 (3.69)	Mental: + 5.2 (9.22)	Physical: + 16.7 (6.61)	–
		Mental: + 4.9 (9.33)	Physical + 16.47 (10.37)	Mental: + 44.26 (9.84)	
	TP	–	–	Physical: + 13.6 (11.66)	–
				Mental: + 2.54	

*AIT* all-inside technique, *TP* transportal technique, *KOOS* knee injury and osteoarthritis outcome score, *IKDC* International Knee Documentation Committee Subjective Knee Form, *KT-1000* the KT-1000 knee arthrometer, *VAS* visual analog scale

### Complications

The pooled data from all the studies showed that the similar complication rates in AIT and TP techniques (8.26 percent vs. 6.62 percent, respectively) – with graft failure, ACL failure, and paresthesia being the most common complications (Table 3). The four studies that directly compare AIT and TP techniques [66–69], on the other hand, showed that three patients in the AIT group had post-operative complications such as ACL failure (*n* = 1), septic arthritis (*n* = 1), and cyclops syndrome (*n* = 1). In the TP group, however, five patients developed complications: ACL failure (*n* = 2), hemarthrosis (*n* = 1), and cyclops syndrome (*n* = 2).

## Discussion

The literature review did not identify a significant difference in post-operative functional outcomes between AIT and TP group. However, post-operative VAS pain scores and complications rates was lower AIT group compared to the TP group in studies directly comparing the two techniques prospectively, suggesting AIT as a good alternative method, especially when treating athletes with ACL injury.

With the increase of ACL reconstruction surgeries worldwide, assessing various techniques is essential to improve patient's long-term functional outcomes by selecting the most suitable method. In this systematic review, we aimed to compare TP-ACLR as a conventional technique with AIT-ACLR as a developing technique through different aspects such as technique-related features and their clinical outcomes. Based on the reviewed literature, AIT and TP technique each has its own advantages and disadvantages; however, AIT is a suitable alternative method considering preserving bony tissue and gracilis tendon with less post-operative pain and complications, along with more knee flexor strength and equal outcomes compared to TP technique. Ultimately, the method of choice is based upon the surgeon’s available equipment; graft choice; experience; efficiency; patient age and activity level; and cosmesis and other relative factors.

An important aspect of ACL reconstruction is the creation of the femoral tunnel. Throughout time, the technique of choice for ACLR has shifted from the transtibial technique to the TP technique, which independently utilizes an anteromedial (AM) arthroscopic portal or an accessory AM portal for anatomic femoral tunnel reconstruction [24, 70]. The accessory AM portal offers numerous advantages including (I) By operating through the AM accessory portal as a viewing portal, we bypass the lateral femoral as a visual obstacle and therefore achieve better femoral tunneling. Also, following the tunnel position is attainable without taking out the drilling device Altering the obliquity of the accessory portal provides establishing femoral tunnel closer to the lateral wall of the notch and therefore disregards the need for notchplasty for visualization and operating [71].

The AM portal is one of the main strengths of the TP technique which allows the surgeon to obtain the optimal setting for ACLR by adjusting the port based on his understanding of the femoral structure and skills [72]. Among the other advantages of this technique is that the horizontally positioning of the graft results in a decrease in rotational instability [71]. Furthermore, the anatomical positioning of the femoral tunnel in the TP technique has resulted in improved stability based on biomechanical and clinical studies; however, the long-term clinical results and ACLR failure are still a matter of debate [18, 46, 73–75].

The difficulty of seating the endoscopic aimer and maintaining the aimer in a hyper-flexed knee is a frequent criticism of the TP technique. Another disadvantage of the TP technique is portal tightening and difficulty viewing in hyperflexion [10]. Moreover, technically challenging short or bicortical sockets, which can limit fixation options, possible damage to the articular cartilage, increased risk of injury to the common peroneal nerve, posterior-wall blowout, increased revision rate, and extension loss during the stance phase are among the other weaknesses of the TP technique that can affect the clinical outcome after ACLR [18, 53, 65, 76, 77]. Furthermore, some studies have shown no definite advantages of the TP-ACLR and its modifications compared to the transtibial-ACLR regarding their clinical outcomes [46, 75, 78, 79].

All-inside technique is considered as a new minimally invasive option for ACLR. The all-inside technique differs from other ACLR approaches in that it uses a "socket" or "half-tunnel" on both the femoral and tibial sides [80, 81]. Reduced incidence of complications such as tibial plateau fractures; more anatomic placement of the tibial tunnel; improved bone-graft integration as a result of manual drilling; improved cosmesis; increased postoperative muscle, tendon, and bone preservation; and improvements in long-term function are among the AIT's proposed benefits [37, 66, 81, 82]. According to Lubowitz, a reduction in postoperative pain can be attributed to a reduction in tibial skin incisions and tibial periosteal irritation [21]. Furthermore, the use of the socket has been proposed to accelerate graft maturation and prevent tunnel enlargement due to dead space elimination [22].

All-inside ACLR technique has some advantages over conventional reconstruction technique that has led to wider use of this technique over the past years. The most noticeable advantage is the elimination of the large incision on the medial side of the tibia required for tibial drilling, which improves the cosmetic aspect [83, 84]. Moreover, creating sockets rather than full tunnels have some benefits including removing fewer bony structures which lead to less post-operative pain and inflammation, along with bone preservation in cases in which subsequent multiple ligament reconstruction is needed in the near future [21, 68, 85]. AIT-ACLR is a promising technique for reliably creating appropriately wide grafts without the requirement for allograft augmentation [86], which can be performed by harvesting a single semitendinosus graft, while also preserving the gracilis tendon. Since the hamstring tendon is considered as a secondary medial stabilizer of the knee and intact gracilis tendon can again be used if additional surgeries are needed; Additionally, gracilis sparing technique is beneficial to functional activity and sports with high demand on hamstring muscle strength [87]. Thus a technique that only harvest semitendinosus tendon seems to be superior to others [88, 89].

There is concern about the windshield wiper and bungee cord phenomenon that may occur with suspensory fixation. Prior studies evaluating sockets drilled with an all-inside ACL technique have revealed less socket expansion and preserved bone stock compared to full tunnels seen in standard ACL techniques on x-ray and CT scans [90, 91]. This is extremely crucial when drilling the tibial socket for all-inside ACL suspensory fixation because it reduces the risk of tibial microfracture trauma seen with full tunnel tibial drilling in standard ACL techniques [92]. Moreover, when closed-sockets are created, there is less graft length available for the windshield-wiper and bungee cord phenomenon compared to full tunnels [22].

Nevertheless, AIT-ACLR is also accompanied by some disadvantages. Adaptation and learning new surgery techniques is always time-consuming and requires practice as some techniques such as graft preparation, fixation, and socket creation involves going through applicable learning courses [22, 83]. Additionally, when creating a socket via retro drill, necessary precautions must be considered to avoid damaging the extra-articular surface [27]. In the aspect of graft fixation, it is reported that suspensory fixation might increase the risk of tunnel widening due to the "windshield wiper" phenomenon [93, 94]. On the other hand, circumferential filling of the socket with the graft might decrease the synovial fluid backflow into the socket and increased bone to graft contact compared to interference screws [30, 95].

The optimal outcome scoring system for evaluating the outcome of ACLR is still a controversial issue, in which various subjective or objective scoring methods such as IKDC, Lysholm, KSS, SF-12, KOOS, and VAS scoring systems were used among the studies. The overall perspective was that there is no significant difference regarding pre-operation and post-operation scores in both AIT and TP ACLR techniques, except regarding the VAS pain score [66–69]. However, there was no difference in narcotic drug consumption in both groups, patients who underwent AIT-ACLR surgery reported lower VAS pain scores and a more rapid decrease in pain in the following months after surgery [66, 68]. Furthermore, Kouloumentas et al. [67] reported a superior knee flexion strength in those who underwent all-inside surgery compared to the conventional group. It is worth mentioning that surgery time in all-inside surgery was longer than TP, which can be explained by the fact that AIT is a new method for surgery and more experience will lead to shorter surgery durations [66, 68]. Regarding post-op complications, AIT demonstrated fewer complications compared to the TP technique in four prospective studies directly comparing the two techniques. As sample sizes and reported complications were few, further studies in this manner are needed to conclude a better decision.

Graft selection and surgical technique during ACL reconstruction have always been a source of contention because they have a direct impact on the outcome. Recent studies, including our systematic review, have shown that AIT is equally effective to TP technique in terms of outcome, with lower pain score and lower mid-term complications, highlighting the advantages of AIT over TP technique in the future. Graft length and thickness, on the other hand, are equally important in achieving good results. Given that the AIT requires quadrupled semitendinosus tendon, it was demonstrated in our review to achieve adequate graft length and thickness. Prior studies has shown that grafts with diameters less than 8 mm have a high graft failure rate [96–99]. Furthermore, grafts of 9 mm in diameter have been shown to reduce graft failure rate by 55% when compared to graft thickness of 7 mm, and grafts of 9 mm or more in diameter have results comparable to patellar tendon graft in ACL reconstruction patients [99, 100]. However, the patient's height has an effect on the semitendinosus graft length and diameter, and a diameter of 8–9 mm may be difficult to achieve with an isolated semitendinosus in some patients, particularly those who are short. Future studies are recommended to evaluate the impact of height on adequacy of isolated semitendinosus graft in patients undergoing ACL reconstruction using AIT.

### Limitations

This study has few limitations that need to be highlighted. Firstly, the modest sample size and fewer number of the comparison studies with studies having relatively short follow-up periods, thus fail to provide long-term clinical evidence. Further comparison and randomized controlled studies with more patients are warranted to evaluate the clinical outcomes and complications of the reported methods. Our review was limited to articles in the English language and focused on prospective clinical trials in order to decrease the chance of bias. Also, articles published after the search period were not included in this review. Moreover, for this review, the commonly used PubMed, Medline, Google Scholar, and EMBASE databases were searched. As a result, the literature that could have aided this study by reviewing other databases such as Cochrane Library, Web of Science, Scopus, SportDiscus, and CINAHL may have been overlooked. Lastly, the analysis was not performed in a blinded fashion, and data in some studies were missing. The use of various outcome measuring methods and missing data leads to the inability to perform a meta-analysis to quantify the overall outcome of the AIT versus TP technique.

## Conclusion

Since the future trend in orthopedic surgery is toward less invasive and patients’ satisfaction with good outcomes, AIT is a good alternative method considering preserving bony tissue and gracilis tendon with less post-operative pain, along with more knee flexor strength and equal outcomes compared to conventional surgery. However, modifications can be applied to improve this technique which requires further comparison studies and evaluations of various grafts, fixations, drilling methods, and outcomes.

## Supplementary Information

**Additional file 1.** Search strategy across online databases.

**Additional file 2.** Worksheet for data extraction of reviewed literature regarding anterior cruciate ligament reconstruction techniques.

## Data Availability

All data generated or analyzed during this study are included in this article [and its supplementary information files].

## Abbreviations

ACL: Anterior cruciate ligament
ACLR: Anterior cruciate ligament reconstruction
AHPLT: Anterior half of peroneus longus tendon
AIT: All-inside techniques
AM: Anteromedial
BPTB: Bone patellar tibial bone
GT: Gracilis tendon
HT: Hamstring tendon
IKDC: International knee documentation committee
MCMS: Modified Coleman Methodology Score
KOOS: Knee injury and osteoarthritis outcome score
KSS: Knee society score
SD: Standard deviation
ST: Semitendinosus
TP: Transportal
TT: Tibial tendon
VAS: Visual analog scale
