# Supplementary material for: Transportal versus all-inside techniques of anterior cruciate ligament reconstruction: a systematic review
Source: J Orthop Surg Res. 2021 Dec 23;16:734. doi: 10.1186/s13018-021-02872-x (PMC8705139; doi:10.1186/s13018-021-02872-x)
Supplement: Supplementary file 1 — Additional file 1. Search strategy across online databases. [file 13018_2021_2872_MOESM1_ESM.docx]

**Additional file 1: Table S1.** Search strategy across online databases

| **Database** | **Medline** | **Embase** | **PubMed** | **Google Scholar** |
| --- | --- | --- | --- | --- |
| **Strategy** | 1. exp anterior cruciate ligament  reconstruction/ | 1. exp anterior cruciate ligament  reconstruction/ | MeSH terms:  ((“anterior cruciate ligament reconstruction”  OR (“anterior cruciate ligament”  OR “posterior cruciate  ligament”))) AND surgery | “Anterior cruciate ligament reconstruction”  OR “anterior cruciate ligament”  AND “All-Inside technique” OR “Transportal Technique” |
|  | 2. exp anterior cruciate ligament/ or  posterior cruciate ligament/ | 2. exp anterior cruciate ligament/ or  posterior cruciate ligament/ |  |  |
|  | 3. 1 or 2 | 3. 1 or 2 |  |  |
|  | 4. surg$.mp | 4. surg$.mp |  |  |
|  | 5. 3 and 4 | 5. 3 and 4 |  |  |
|  | 6. limit 5 to (human and English  language) | 6. limit 5 to (human and English  language) |  |  |

Search strategy. A total of 14627 articles were found in a PubMed search, 12777 were found in an Embase search, and 9455 were found in a Medline

search to give a total of 36,859.
