# Supplementary material for: Transportal versus all-inside techniques of anterior cruciate ligament reconstruction: a systematic review
Source: J Orthop Surg Res. 2021 Dec 23;16:734. doi: 10.1186/s13018-021-02872-x (PMC8705139; doi:10.1186/s13018-021-02872-x)
Supplement: Supplementary file 2 — Additional file 2. Worksheet for data extraction of reviewed literature regarding anterior cruciate ligament reconstruction techniques. [file 13018_2021_2872_MOESM2_ESM.docx]

**Additional file 2: Table 2.** Worksheet for data extraction of reviewed literature regarding anterior cruciate ligament reconstruction techniques

| N. | Variable |
| --- | --- |
|  | Author |
|  | Country |
|  | Year |
|  | Study type |
|  | Number of patients |
|  | Male: Female |
|  | Mean age (+SD) |
|  | BMI |
|  | Injury to surgery interval (mean +SD weeks) |
|  | Mechanism of injury |
|  | Population |
|  | Follow up (mean) |
|  | Early follow up |
|  | Mid follow up |
|  | Late follow up |
|  | Surgical technique |
|  | Modified |
|  | Graft type |
|  | Hamstring |
|  | Semitendinosus |
|  | Gracilis |
|  | Quadriceps |
|  | Achilis |
|  | BTBP |
|  | Augmentation |
|  | Mixed |
|  | Other |
|  | Mean graft length |
|  | Mean graft diameter |
|  | Autograft |
|  | Allograft |
|  | Type of graft spinning |
|  | Femoral Drilling technique |
|  | Tibial Drilling technique |
|  | Femoral socket range (mm) |
|  | Tibial socket range (mm) |
|  | Graft Fix Tibial side |
|  | Graft fixation femoral side |
|  | Rehabilitation |
|  | Early |
|  | Late |
|  | Return to sport |
|  | Jogging |
|  | Outcome |
|  | Pre op - Knee injury and osteoarthritis outcome score (KOOS) |
|  | Post op - Knee injury and osteoarthritis outcome score (KOOS) |
|  | KOOS change after 2 years |
|  | pre op – Lachman |
|  | Post op -Lachman |
|  | Lachman change after 2 years |
|  | pre op - Subjective international knee documentation committee (IKDC) |
|  | post op - Subjective international knee documentation committee (IKDC) |
|  | Subjective international knee documentation committee (IKDC) change after 2 years |
|  | Pre op - Pivot shift |
|  | Post op - Pivot shift |
|  | Pivot shift change after 2 years |
|  | Pre op KT-1000 Post op KT-1000 |
|  | KT-1000 change after 2 years |
|  | Pre op - Lysholm Score |
|  | Post op - Lysholm score |
|  | Lysholm score change after 2 years |
|  | Pre op- Visual analog scale (VAS) |
|  | Post op- Visual analog scale (VAS) |
|  | Visual analog scale (VAS) change after 2 years |
|  | Pre op - Tegner Activity Score |
|  | Post op - Tegner Activity Score after 2 years |
|  | Tegner activity score change after 2 years |
|  | Pre op- Short form 12 physical |
|  | Post op- Short form 12 physical |
|  | pre op SF-12 mental Scores |
|  | post op SF-12 mental Scores |
|  | Short form 12 after 2 years |
|  | Pre op - Side to side laxity |
|  | Post op - Side to side laxity |
|  | Side to side laxity after 2 years |
|  | Pre op- Knee society score (KSS) pain |
|  | Post op- Knee society score (KSS) pain |
|  | Pre op- Knee society score (KSS) function |
|  | Post op- Knee society score (KSS) function |
|  | Knee society score (KSS) after 2 years |
|  | Average knee flexion at follow up |
|  | Flexion deficit after 9 months |
|  | Extension Lag after 2 year |
|  | Pre-op one leg hop test post-op one leg hop test |
|  | one leg hop test change after 2 years |
|  | pre-op patelofemoral pain score |
|  | post-op patelofemoral pain score |
|  | patelofemoral pain score after 2 years |
|  | Total Complication |
|  | ACL failure |
|  | Graft failure |
|  | Extension loss |
|  | Flexion loss |
|  | Cartilage damage |
|  | Meniscus injury |
|  | Neurapraxia |
|  | wound dehiscence |
|  | septic arthritis |
|  | Arthrofibrosis |
|  | Hypoesthesia |
|  | Paresthesia |
|  | Superficial infection |
|  | Deep Infection |
|  | Superficial haematoma |
|  | Hemarthrosis |
|  | Thromboembolism |
|  | Cyclops syndrome |
|  | Tunnel widening |
|  | peronel nerve palsy |
|  | Reoperation |
|  | Post-op pain |
|  | Cost |
